# Pathogenesis of Korean *Sapelovirus* *A* in piglets and chicks

**DOI:** 10.1099/jgv.0.000571

**Published:** 2016-10-13

**Authors:** Deok-Song Kim, Mun-Il Kang, Kyu-Yeol Son, Geon-Yong Bak, Jun-Gyu Park, Myra Hosmillo, Ja-Young Seo, Ji-Yun Kim, Mia Madel Alfajaro, Mahmoud Soliman, Yeong-Bin Baek, Eun-Hyo Cho, Ju-Hwan Lee, Joseph Kwon, Jong-Soon Choi, Ian Goodfellow, Kyoung-Oh Cho

**Affiliations:** ^1^​ Laboratory of Veterinary Pathology, College of Veterinary Medicine, Chonnam National University, Gwangju, Republic of Korea; ^2^​ Division of Virology, Department of Pathology, University of Cambridge, Addenbrooke’s Hospital, Cambridge, UK; ^3^​ Chonnam National University Veterinary Teaching Hospital, Gwangju, Republic of Korea; ^4^​ Division of Life Science, Korea Basic Science Institute, 169-148 Gwahak-ro, Yuseong-gu, Daejeon 305-806, Republic of Korea

**Keywords:** *Sapelovirus A*, piglets, chicks, pathogenesis, host range restriction

## Abstract

*Sapelovirus A* (SV-A), formerly known as porcine sapelovirus as a member of a new genus *Sapelovirus*, is known to cause enteritis, pneumonia, polioencephalomyelitis and reproductive disorders in pigs. We have recently identified α2,3-linked sialic acid on GD1a ganglioside as a functional SV-A receptor rich in the cells of pigs and chickens. However, the role of GD1a in viral pathogenesis remains elusive. Here, we demonstrated that a Korean SV-A strain could induce diarrhoea and intestinal pathology in piglets but not in chicks. Moreover, this Korean SV-A strain had mild extra-intestinal tropisms appearing as mild, non-suppurative myelitis, encephalitis and pneumonia in piglets, but not in chicks. By real-time reverse transcription (RT) PCR, higher viral RNA levels were detected in faecal samples than in sera or extra-intestinal organs from virus-inoculated piglets. Immunohistochemistry confirmed that high viral antigens were detected in the epithelial cells of intestines from virus-inoculated piglets but not from chicks. This Korean SV-A strain could bind the cultured cell lines originated from various species, but replication occurred only in cells of porcine origin. These data indicated that this Korean SV-A strain could replicate and induce pathology in piglets but not in chicks, suggesting that additional porcine-specific factors are required for virus entry and replication. In addition, this Korean SV-A strain is enteropathogenic, but could spread to the bloodstream from the gut and disseminate to extra-intestinal organs and tissues. These results will contribute to our understanding of SV-A pathogenesis so that efficient anti-sapelovirus drugs and vaccines could be developed in the future.

## Introduction

The *Picornaviridae* family, comprising 29 genera, consists of a diverse family of non-enveloped viruses with positive-sense single-stranded RNA genomes (Racaniello, 2013; http://talk.ictvonline.org/files/master-species-lists/m/msl/5208). Viruses in this family can cause a wide range of diseases, including intestinal, respiratory, neurological, cardiac, hepatic, mucocutaneous and systemic diseases of various severities in both humans and animals (Racaniello, 2013). Since porcine enterovirus 8 (PEV-8), simian type 2 picornavirus and duck picornavirus TW90A have a unique genomic organization different from other picornavirus genera (Son *et al.*, 2014a), the genus *Sapelovirus* is a newly assigned member of the *Picornaviridae* family (Adams *et al.*, 2015). The *Sapelovirus* genus consists of three species: *Sapelovirus A* (SV-A) formerly known as porcine sapelovirus, *Sapelovirus B* formerly named as simian sapelovirus and *Avian sapelovirus* formerly known as duck picornavirus TW90A (Adams *et al.*, 2015).

SV-A can cause asymptomatic and symptomatic diseases in both field and experimental pigs (Alexandersen *et al.*, 2012; Kim *et al.*, 2016). The symptomatic disorders include diarrhoea, pneumonia, polioencephalomyelitis and reproductive disorders (Alexandersen *et al.*, 2012; Huang *et al.*, 1980; Lamont & Betts, 1960; Lan *et al.*, 2011; Schock *et al.*, 2014; Sibalin, 1963). Experimental studies have demonstrated a diverse range of clinical symptoms (Lamont & Betts, 1960; Lan *et al.*, 2011; Sibalin, 1963; Yamanouchi *et al.*, 1965). These differences largely depend on age, route of infection and strains inoculated (Alexander & Betts, 1967; Lamont & Betts, 1960; Lan *et al.*, 2011; Sibalin, 1963; Yamanouchi *et al.*, 1965).

Significant antigenic diversity has been observed in SV-As isolated from different countries and continents (Bohl *et al.*, 1960; Dunne *et al.*, 1967, 1971; Izawa *et al.*, 1962; Kadoi *et al.*, 1970; L’Ecuyer & Greig, 1966). The genomes of SV-As and other members in the genus *Sapelovirus* have the typical picornavirus genome organization: 5'-UTR-L-VP4-VP2-VP3-VP1-2A-2B-2C-3A-3B-3C-3D-3'-UTR (Krumbholz *et al.*, 2002; Oberste *et al.*, 2002, 2003; Son *et al.*, 2014a; Tseng & Tsai, 2007). However, there are significant structural differences in the SV-A genome, e.g. the *cis*-acting RNA element (*CRE*) in the 2c coding region and kissing domain in the 3'-UTR vary between recent Korean and Chinese strains and older English and Chinese strains (Son *et al.*, 2014a). These differences in antigenic diversity and structural features can influence the pathogenicity and/or host range restriction of SV-A strains, yet we have a limited understanding of the pathogenesis of SV-A (Son *et al.*, 2014a).

In comparison to other picornaviruses, the SV-A life cycle remains poorly characterized. We have recently demonstrated that SV-A can recognize a2,3-linked sialic acid (SA) on GD1a as a functional SV-A receptor (Kim *et al.*, 2016). a2,3-linked SA is known to be highly expressed on cells of porcine and avian origin (de Graaf & Fouchier, 2014; Raman *et al.*, 2014), indicating that SV-A has the potential to infect both pigs and chickens. Therefore, the objective of this study was to undertake a comparative analysis of the pathogenesis of a Korean SV-A strain in piglets and chicks.

## Results

### SV-A strain caused diarrhoea and faecal viral shedding in piglets but not in chicks

Chicken cells are typically rich in α2,3-linked SA, whereas porcine cells are abundant in both α2,3- and α2,6-linked SAs (Raman *et al.*, 2014). Our previous results have demonstrated that SV-A could recognize α2,3-linked SA as a receptor (Kim *et al.*, 2016), suggesting that SV-A might be able to infect and induce pathology in both pigs and chicks. To determine whether SV-A could induce diarrhoea and faecal viral shedding in piglets and chicks, 3-day-old piglets obtained from sows by hysterectomy and 3-day-old specific pathogen-free (SPF) chicks were orally inoculated with 2×10^9^ or 5×10^8^ p.f.u. ml^−1^ of SV-A (KS04105 strain), respectively. Compared to mock inoculation, piglets inoculated with SV-A strain had continuous diarrhoea from 1 to 5 days post-inoculation (dpi) (data not shown). However, diarrhoea was not observed in mock- or SV-A-inoculated chicks during the entire experimental period.

**Fig. 1.**
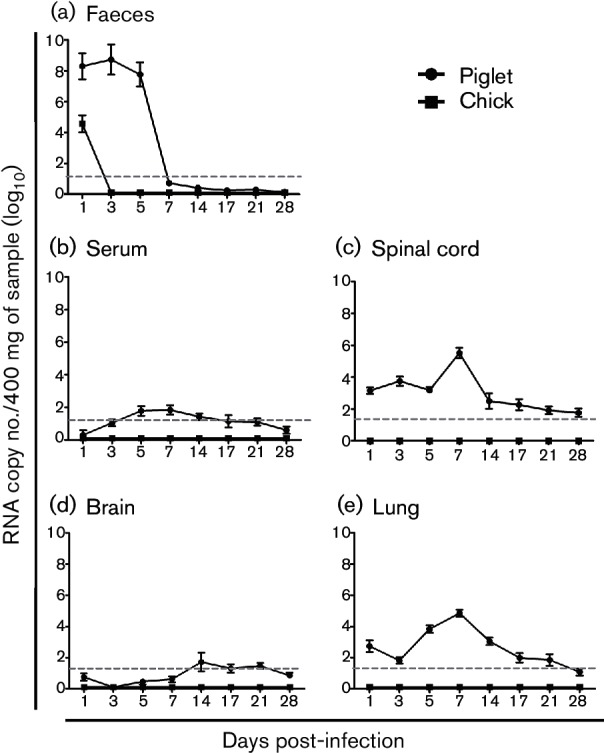
SV-A viral RNA levels in faeces (a), serum (b), spinal cord (c), brain (d) and lung (e) samples obtained from SV-A-inoculated piglets and chicks were determined by SYBR Green real-time RT-PCR. All experiments were performed three independent times. Error bars indicate sd from triplicate experiments. Dashed line indicates the limit of detection.

To assess faecal viral shedding, one-step real-time quantitative reverse transcription PCR (qRT-PCR) assay was performed with faecal samples sequentially collected from mock- or SV-A-inoculated piglets and chicks (Chen *et al.*, 2014). High viral RNA levels were detected in faecal samples collected from piglets at 1 dpi, reaching a peak at 3 dpi followed by decreasing viral loads from 5 dpi (Fig. 1a). However, SV-A RNA was only detected at 1 dpi from faeces of SV-A-inoculated chicks (Fig. 1b), most likely representing virus inoculum passing through the intestines. Collectively, these data indicated that SV-A could induce diarrhoea and faecal shedding in piglets but not in chicks.

### SV-A strain caused intestinal and extra-intestinal lesions in piglets but not in chicks

**Fig. 2.**
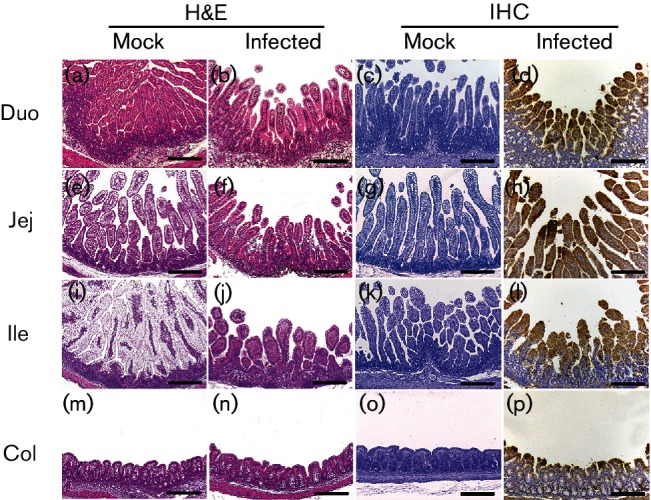
Histological changes and antigen distribution in the intestine of piglets inoculated with or without SV-A. Small- and large-intestinal tissues collected from mock- or SV-A-inoculated piglets were examined histopathologically and immunohistochemically. Bars, 200 µm. Duo, duodenum; Jej, jejunum; Ile, ileum; Col, colon.

**Fig. 3.**
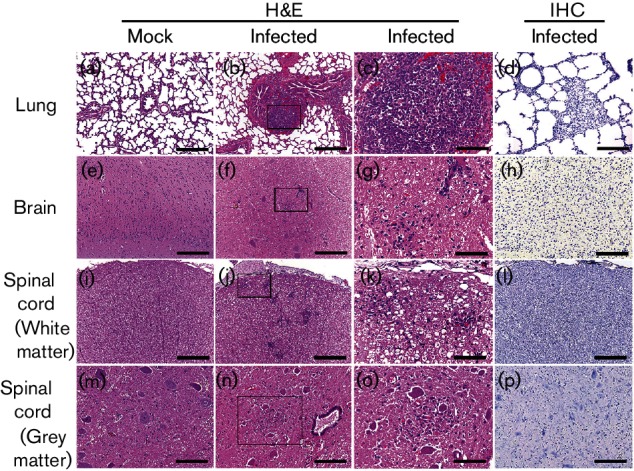
Histological changes and antigen distribution in extra-intestinal organs and tissues of piglets inoculated with or without SV-A. Lung, brain and spinal cord samples collected from mock- or SV-A-inoculated piglets were examined histopathologically and immunohistochemically. (c), (g), (k) and (o) represent higher magnifications of samples shown in (b), (f), (j) and (n), respectively. Bars, 200 µm (a, b, d, e, f, h, i, j, l and p), 100 µm (m and n), 50 µm (c, g, k and o).

**Fig. 4.**
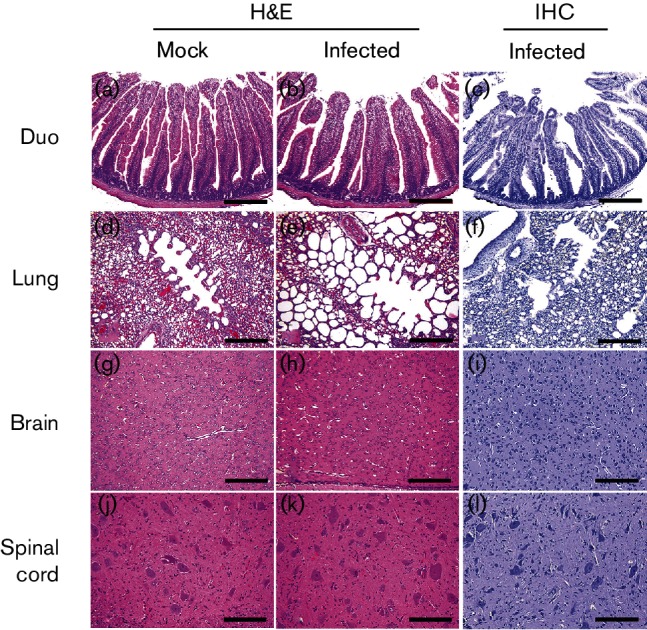
Histological changes and antigen distribution in intestinal and extra-intestinal organs and tissues collected from chicks inoculated with or without SV-A. Intestinel, lung, brain and spinal cords isolated from mock- or SV-A-inoculated chicks were examined histopathologically and immunohistochemically. Bars, 100 µm (a, b, d, e, g, h, j and k), 50 µm (c, f, i and l). Duo, duodenum.

We then assessed the histopathological changes in organs and tissues sequentially sampled from mock- or SV-A- inoculated piglets and chicks. SV-A infection resulted in histopathological changes in the small intestines of virus-inoculated piglets, including villous atrophy and crypt hyperplasia at 1 dpi (Table 1). These mucosal changes were gradually increased in all regions of the small intestine until 5 dpi followed by a decrease at 7 dpi (Fig. 2, Table 1). Large intestinal lesions, including crypt fusion with epithelial cell hyperplasia, were observed at 2 dpi, increased until 5 dpi followed by a decrease at 7 dpi (Fig. 2). Lungs from infected piglets showed lymphoid cell infiltration in the peribronchiolar submucosa and perivascular space from 5 dpi to the end of the experiment (Fig. 3). As shown in Table 2, adaptive immune reactions to SV-A infection evident as perivascular cuffing of lymphocytes and gliosis were observed in both grey and white matters of spinal cord (myelitis) and brain (encephalitis) (Fig. 3). These typical host defence reactions were observed from 7 dpi until the termination of the experiment. Neuronophagia and chromatolysis were not frequently found in the spinal cord or the brain. However, spinal cords showed stronger inflammatory reactions than the brain (Table 2). No specific lesion was observed in other organs or tissues collected from piglets regardless of SV-A infection. However, SV-A did not induce any histopathological change in any organ or tissue sequentially sampled from SV-A-inoculated chicks during the entire experimental period (Fig. 4). These data indicated that SV-A could induce pathology in piglets but not in chicks.

**Table 1.** Summary of histopathological findings in the intestine of colostrum-deprived piglets after inoculation with SV-A strain KS04105

Piglet no.	dpi at euthanasia	Duodenum		Jejunum		Ileum	
		Lesion score*	Antigen distribution†	Lesion score	Antigen distribution	Lesion score	Antigen distribution
1–3	1	3.6±0.53	3.3±0.61	2.1±0.4	2.4±0.55	2.3±0.58	1.6±0.51
4–6	3	3.6±0.36	3±1.11	3.4±0.51	2.7±0.58	2.8±0.7	2.3±0.42
7–9	5	2.9±0.47	1.2±0.35	4±0.29	1.8±0.4	3.3±0.46	1.3±0.25
10–12	7	2.3±0.89	0	3.2±0.75	0	2.1±0.65	0
13–15	14	0	0	0	0	0	0
16–18	17	0	0	0	0	0	0
19–21	21	0	0	0	0	0	0
22–24	28	0	0	0	0	0	0
25–27‡	5	0	0	0	0	0	0

*Intestinal changes were scored according to the average villi/crypt (V/C) ratio plus the grade of epithelial cell desquamation as follows: V/C ratio: 0, normal (V/C≥6 : 1); 1, mild (V/C=5.0–5.9 : 1); 2, moderate (V/C=4.0–4.9 : 1); 3, marked(V/C=3.0–3.9 : 1); 4, severe (V/C≤3.0 : 1). Desquamation grade: 0, normal (no desquamation); 1, mild (cuboidal attenuation of tip villous epithelium); 2, moderate (desquamation of upper villous epithelium); 3, marked (desquamation of lower villous epithelium); 4, severe (desquamation of crypt epithelium).

†Antigen distribution in the small intestine was evaluated based on the number of antigen-positive cells in the villi as follows: 0, no positive cells; 1, one to two positive cells in the villi; 2, three to five positive cells scattered in the villi; 3, many positive cells in the villi; 4, positive in almost all epithelial cells in the tip and upper part of the villi.

‡Mock inoculation with serum-free EMEM.

**Table 2.** Summary of histopathological findings in the brain and spinal cord of colostrum-deprived piglets after inoculation with SV-A strain KS04105

Piglet no.	dpi at euthanasia	Brain		Spinal cord	
		Lesion score*	Antigen distribution†	Lesion score	Antigen distribution
1–3	1	0	0	0	0
4–6	3	0	0	0	0
7–9	5	0	0	0	0
10–12	7	1±1	0	0.7±0.58	0
13–15	14	1.3±0.58	0	2.3±0.58	0
16–18	17	1.7±0.58	0	1.7±0.58	0
19–21	21	1.3±0.58	0	2.3±0.57	0
22–24	28	1.7±0.58	0	1.3±0.58	0
25–27‡	5	0	0	0	0

*Histopathological changes in the central nervous system were scored according to the severity of gliosis and perivascular cuffing as follows: 0, normal; 1, one or two glioses or perivascular cuffing; 2, ≥2, ≤3 glioses and/or perivascular cuffing; 3, multiple glioses and/or perivascular cuffing with heavy infiltration of lymphocytes or glial cells.

†Antigen distribution in the central nervous system was evaluated based on the number of antigen-positive cells as follows: 0, no positive cells; 1, one to two positive cells; 2, three to five positive cells; 3, many positive cells.

‡Mock inoculation with serum-free EMEM.

### Viral antigen was detected in the intestine of piglets but not chicks

To assess the distributions of SV-A antigen in the organs and tissues, immunohistochemical assay was performed with organs and tissues sampled sequentially from mock- or SV-A-infected piglets and chicks using monoclonal antibody (mAb) specific to SV-A capsid protein. SV-A antigen was only detected in the epithelial cells of villi from SV-A-infected piglets at 1 to 5 dpi (Fig. 2, Table 1). Other organs and tissues collected from mock- or SV-A-inoculated piglets were negative for SV-A antigen (Fig. 3, Table 2). Consistent with clinical and histopathological observations, SV-A antigen was not detected in any organ or tissue collected from SV-A-inoculated chicks (Fig. 4).

### SV-A caused viraemia and replicated in extra-intestinal organs of piglets but not chicks

To assess whether SV-A induced viraemia and replicated in extra-intestinal organs of piglets and chicks, qRT-PCR assay was performed with sera and extra-intestinal organs and tissues collected from mock- or SV-A-inoculated piglets and chicks. SV-A RNA levels were relatively low in the serum, spinal cord, lung and brain in comparison with those in the faecal samples collected from SV-A-inoculated piglets (Fig. 1a). However, SV-A RNA was not detected in the sera and any extra-intestinal organs and tissues collected from mock- or SV-A-inoculated chicks. These data indicated that SV-A induced viraemia, which was then disseminated to extra-intestinal organs and tissues in piglets but not chicks.

### Binding and infection abilities of SV-A to various cells

**Fig. 5.**
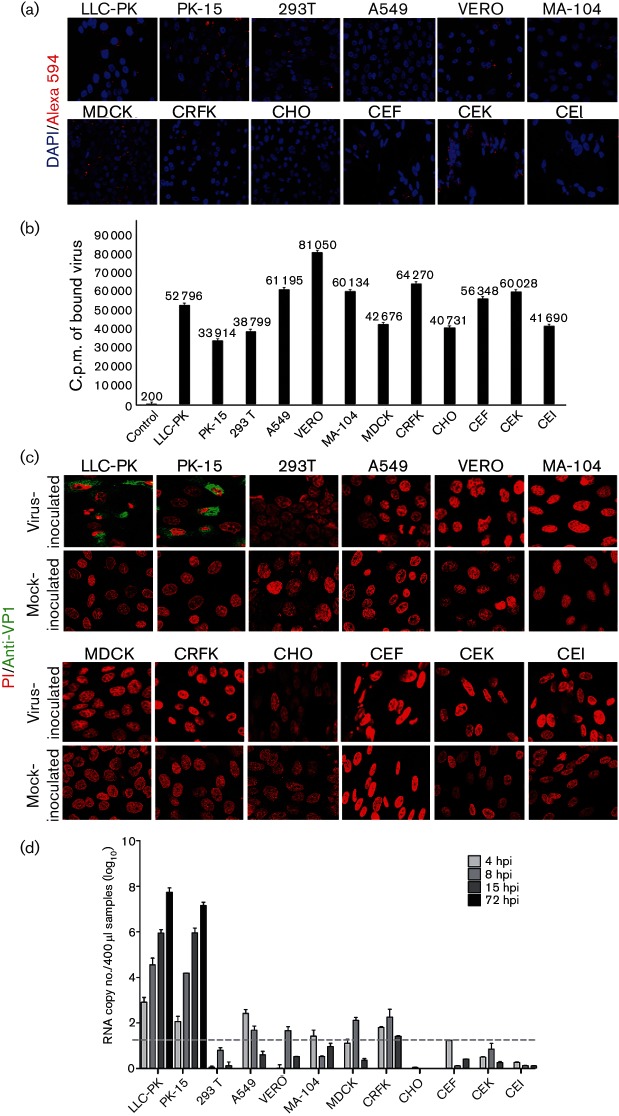
SV-A binds to and infects cells of porcine origin. (a) Binding of AF-594-labelled mock or SV-A (50 000 c.p.m.) to various cells from different species was observed by confocal microscopy. (b) Binding of [^35^S]methionine/cysteine-labelled mock or SV-A (50 000 c.p.m.) to various cells from different species was measured by liquid scintillation counting. (c) Infectivity of SV-A to various cells from different species was determined by immunofluorescence assay using a mouse mAb against SV-A VP1 protein at 15 h post-infection. (d) Quantification of SV-A genome copy numbers in various cells from different species was determined by SYBR Green real-time RT-PCR. All experiments were performed independently three times. Error bars indicate sd from triplicate experiments. Dashed line indicates the limit of detection.

To determine whether SV-A had a strict tropism for porcine cells *in vitro*, the binding and infection ability of SV-A was examined with various cell lines, including porcine, human, chicken embryo, canine, simian, hamster and feline. Alexa Fluor 594 (AF-594)-labelled SV-A was attached to all cell lines at various degrees (Fig. 5a). Radio-labelled SV-A was able to bind to all cells examined at similar degrees (Fig. 5b). However, SV-A was only replicated in cells of porcine origin (LLC-PK and PK-15) (Fig. 5c) with similar levels of cytopathic effect (data not shown). The SV-A genome copy numbers robustly increased in cells of porcine origin in a time-dependent manner, but not in cells of other species (Fig. 5d). Our results indicated that only porcine cells were available for SV-A infection.

## Discussion

All viruses initiate infection by binding to specific receptor(s) on the surface of susceptible host cells (Neu *et al.*, 2011). We have previously demonstrated that SV-A could utilize α2,3-linked SA on GD1a glycolipid as a receptor (Kim *et al.*, 2016). Indeed, glycolipid-associated α2,3-terminal SA is abundant on the cell surface of both avian and porcine species (de Graaf & Fouchier, 2014; Raman *et al.*, 2014). However, our results in this study revealed that SV-A infection was limited to cells of porcine origin. It could not infect cells of other origins including chickens and humans. Moreover, SV-A was replicated in piglets but not in chicks, confirming that SV-A could not cause interspecies transmission at least between pigs and chickens. Our results also suggested that other factors in addition to glycolipid-associated α2,3-terminal SA might be required for efficient SV-A replication in cells of non-porcine origin. We have previously observed that chymotrypsin or trypsin treatment of cells has no effect on virus infection (Kim *et al.*, 2016), suggesting that cell surface-associated proteins do not play accessory roles in SV-A infection. The nature of post-binding block to infection in cells from non-porcine origin is as yet unknown, but it is possible the SV-A infection of avian cells induces the innate response leading to the restriction of viral replication.

Although previous studies have indicated that SV-A can cause enteritis, pneumonia, polioencephalomyelitis and reproductive disorders (Alexandersen *et al.*, 2012; Huang *et al.*, 1980; Lamont & Betts, 1960; Lan *et al.*, 2011; Schock *et al.*, 2014; Sibalin, 1963), the pathogenicity and/or host range restriction of SV-A have been poorly characterized. In the current study, the most significant lesions were found in the intestines during the early infection period, where severe villous atrophy was found to be associated with high viral RNA loads in the faecal samples and strong SV-A antigen reactivity in the intestinal epithelial cells. In contrast, the spinal cord and brain mainly showed signs of adaptive immune response (non-suppurative inflammation) and comparatively low viral RNA copy numbers without clear evidence of SV-A antigen-positive cells. These data indicated that the Korean SV-A strain (KS04105) used in this study was enteropathogenic. Contrary to our data, a recent study has reported that neuroinvasive English SV-A strain replication in the spinal cord could lead to severe adaptive immune responses (non-suppurative polioencephalomyelitis) without causing lesions in other tissues (Schock *et al.*, 2014). This English SV-A strain (G5) is phylogenetically closer to the English V13 strain than to other SV-A strains circulating recently in Korea and China (Schock *et al.*, 2014; Son *et al.*, 2014a). The molecular basis involved in the differences in virulence and tropism among SV-A strains remains unknown. However, there is clear evidence showing that variation in RNA structures can contribute to viral virulence (Son *et al.*, 2014a). An additional explanation for discordance in tropism may be variation in the age of animals used in studies. The English SV-A study used adult pigs (Schock *et al.*, 2014), whereas this study used neonatal piglets. Further studies are required to examine the predilections of age and inoculation routes, as well as antigenic and genomic differences, so that our understanding of variations in SV-A pathogenesis and/or host range restriction can be improved.

Similar to other enteric viruses (Blutt & Conner, 2007; Park *et al.*, 2007), SV-A RNA loads were detected in the sera of piglets orally inoculated with SV-A strain in this study, suggesting that SV-A might be able to penetrate the gut barrier from the luminal side through destruction of enterocytes in the villi. This result also implied that SV-A could reach other organs and tissues via cell-free transmission. However, the mechanism by which SV-A reaches the blood and spreads to other organs and tissues remains to be determined.

In conclusion, this study demonstrated that the Korean SV-A strain could be replicated and induce pathology in piglets but not chicks. Our data also indicated that this Korean SV-A strain could reach the bloodstream from the gut, and be disseminated to extra-intestinal organs and tissues. These results will improve our understanding of the life cycle and pathogenesis of sapelovirus so that affordable, useful and efficient drugs can be developed for anti-sapelovirus therapy.

## Methods

### Cells and viruses.

LLC-PK, PK-15 and human cervical cancer HeLa cells [American Type Culture Collection (ATCC)] were maintained in Eagle’s minimal essential medium (EMEM) supplemented with 10 % FBS, 100 U ml^−1^ penicillin and 100 µg ml^−1^ streptomycin. Chinese hamster ovary (CHO) cells were kindly provided by Dr Sun-Young Im, Chonnam National University, South Korea. Crandall Reese feline kidney (CRFK), Madin–Darby canine kidney (MDCK), African green monkey kidney Vero and MA-104, human embryonic kidney 293T (HEK293T) and human lung adenocarcinoma cell line A549 cells (ATCC) were grown in Dulbecco’s modified Eagle’s medium (DMEM) supplemented with 5 % FBS, 100 U ml^−1^ penicillin and 100 µg ml^−1^ streptomycin. MA-104 cells (ATCC) were cultured in alpha minimum essential medium supplemented with 5 % FBS, 100 U ml^−1^ penicillin and 100 µg ml^−1^ streptomycin. Human lung fibroblast WI-38 cells (ATCC) were maintained in DMEM supplemented with 5 % FBS, 100 U ml^−1^ penicillin and 100 µg ml^−1^ streptomycin. Primary chicken embryo cells [including primary chicken embryo fibroblast (CEF), kidney (CEK) and intestine (CEI) cells from 12-day-old SPF White Leghorn foetus] were grown in M199 medium supplemented with 10 % FBS, 100 U ml^−1^ penicillin and 100 µg ml^−1^ streptomycin.

SV-A strain KS04105 used in this study was isolated from faecal samples of diarrhoeic piglets from South Korea (Son *et al.*, 2014a, b). This strain was passaged eight times in LLC-PK cells, including isolation, adaptation and triple plaque purification. Isolated viruses were confirmed as PSVs based on immunofluorescence assay (IFA), RT-PCR and transmission electron microscopy (Kim *et al.*, 2016; Son *et al.*, 2014a, b).

### Reagents and antibodies.

AF-594 succinimidyl ester, purchased from Molecular Probes (catalogue number, A-20004), was dissolved in DMSO. The mAb against SV-A capsid protein was kindly provided by Dr M. Dauber (Friedrich Loeffler Institute, Germany). FITC goat anti-mouse IgG antibody and peroxidase-conjugated goat anti-mouse IgG antibody were purchased from Santa Cruz Biotechnology. HRP-conjugated streptavidin was obtained from Jackson Immuno Research Lab. SlowFade Gold Antifade Reagent (Invitrogen) containing DAPI was purchased from Invitrogen.

### Animal experiments.

To evaluate the pathogenicity and host range restriction of SV-A, 3-day-old piglets (*n*=27) from sows by hysterectomy and 3-day-old SPF White Leghorn chicks (*n*=27) were used. Twenty-four piglets and 24 chicks were orally inoculated with 20 ml (1×10^8^ p.f.u. ml^−1^) or 5 ml (1×10^8^ p.f.u. ml^−1^) of the KS04105 strain individually. As negative controls, piglets and chicks were inoculated with the same volume of medium for mock infection. All animals were fed with sterilized commercialized milk or feed. After inoculation, clinical signs including diarrhoea, pneumonia and convulsions were evaluated daily as described previously (Park *et al.*, 2013). Animals were euthanized at specified times (Table 1).

Necropsy was immediately performed after euthanasia. During necropsy, organs and tissues including each intestinal segment, spinal cords, brain, lung and liver were excised from piglets or chicks. They were immediately placed in 10 % buffered formalin for histological examination. Formalin-fixed samples were embedded in paraffin, sectioned, stained with Mayer’s haematoxylin and eosin and examined microscopically (Park *et al.*, 2013). All samples collected for qRT-PCR analysis were immediately snap-frozen in liquid nitrogen and kept at −80 °C until use.

### Immunohistochemistry.

The distribution of SV-A antigens in tissues was evaluated by immunohistochemical examination using paraffin-embedded sections and an mAb against SV-A capsid protein as described previously (Park *et al.*, 2007). Briefly, paraffin-embedded sections of each organ and tissue were deparaffinized and rehydrated through a graded series of alcohol in 0.1 M PBS and then treated with 0.1 % trypsin/0.1 % calcium chloride in PBS for 1 h at 37 °C. Trypsinized sections were first treated with 3 % H_2_O_2_ to quench endogenous peroxidase and then incubated with an mAb against SV-A capsid protein at 4 °C overnight. All sections were stained with peroxidase-labelled streptavidin–biotin (SAB-PO) using a Histofine SAB-PO kit for the mouse mAb. Antigen localization was visualized by incubating the sections with 3,3′-diaminobenzidine/H_2_O_2_ solution. The sections were then weakly counterstained with haematoxylin. To calculate the number of antigen-positive cells in the organs or tissues, 10 fields per section were analysed using a ×40 objective and a ×10 eyepiece, yielding a final magnification of ×400.

### Virus purification by caesium chloride gradient centrifugation.

SV-A strain KS04105 grown in LLC-PK cells was purified using caesium chloride (CsCl) gradient centrifugation as described previously (Kim *et al.*, 2016). Briefly, infected cell cultures were harvested at 72 h post inoculation and freeze-thawed three times. Cell debris was spun down at 2469 ***g*** for 10 min at 4 °C. A total of 500 ml of virus-containing supernatants was concentrated by centrifugation at 245 853 ***g*** for 20 h at 4 °C using a SW40 rotor (Beckman). The viruses in the pellets were resuspended in TNE buffer (50 mM Tris/HCl, 100 mM NaCl and 100 mM EDTA, pH 7.5). The suspension was then layered over discontinuous CsCl gradients. After ultracentrifugation, the virus band was collected by puncturing the side of the tube with a needle. The virus solution was then diluted in distilled water and further purified by ultracentrifugation. Purified viruses were dialysed in 0.1 M sodium bicarbonate buffer (pH 8.3) for fluorescence labelling or in TNE buffer for radioactivity assay overnight. Purified viruses were then stored in aliquots at –80 °C.

### Labelling of viruses with AF-594.

Labelling of viruses with AF-594 was performed as described previously (Kim *et al.*, 2016). Briefly, purified virus (10 mg at 1 mg ml^−^^1^) in 0.1 M sodium bicarbonate buffer (pH 8.3) was labelled with one-tenth-fold molar concentration of AF-594 succinimidyl ester (1 mg at 1 mg ml^−^^1^ in DMSO). Each reaction was mixed thoroughly by vortexing for 30 s and incubated at room temperature for 1 h with continuous stirring. Labelled virus was repurified with CsCl as described above, dialysed against virion buffer and stored in 2 µg aliquots at –20 °C.

### Dye-labelled binding assay.

Dye-labelled binding assay was performed with purified AF-594-labelled viruses as described previously (Kim *et al.*, 2016). Briefly, mock-infected or treated cells were inoculated with m.o.i. of 1000 of AF-594-labelled virus and incubated on ice for 5 min followed by incubation at room temperature for 10 min. Cells were washed extensively with cold PBS, fixed with 4 % formaldehyde and washed three times with cold PBS. Dishes were mounted with SlowFade Gold Antifade Reagent containing DAPI solution for nucleus staining. Infected cells were observed under an LSM 510 confocal microscope and analysed using LSM software (Carl Zeiss).

### Labelling of viruses with [^35^S]methionine/cysteine.

Labelling of viruses with [^35^S]methionine/cysteine (PerkinElmer) was carried out as described previously (Kim *et al.*, 2016). Briefly, confluent monolayers of cells were infected with SV-A strain KS04105 at an m.o.i. of 0.1 p.f.u. cell^−1^ at 37 °C for 4 h. The medium was replaced with RPMI 1640 lacking methionine and cysteine (Sigma-Aldrich). After starving cells for 2 h, they were supplemented with 1 Mbq [^35^S]methionine/cysteine ml^−^^1^ (PerkinElmer). At 72 h following virus infection, each labelled virus was purified by CsCl density gradient centrifugation as described above.

### Binding assay of [^35^S]methionine/cysteine-labelled virus to various cell lines.

Binding of [^35^S]methionine/cysteine-labelled virus to various cell lines was assayed as described previously (Kim *et al.*, 2016). Briefly, cells (4×10^4^) were plated into 96-well plates. Purified [^35^S]methionine/cysteine-labelled virus (50 000 c.p.m.) was incubated with cells on ice for 45 min. Cells were washed three times with ice-cold PBS followed by cell lysis with 0.1 % SDS and 0.1 M NaOH. Total radioactivity in the cell lysate was determined by liquid scintillation counting.

### Infectivity assay.

Infectivity assay of SV-A strain KS04105 in various cell lines was carried out as described previously (Kim *et al.*, 2016). Briefly, confluent monolayers of each cell line on a confocal dish were infected with SV-A strain KS04105 at an m.o.i. of 1 p.f.u. cell^−1^ and incubated at 37 °C for 1 h. Cells were washed three times with PBS and replaced with maintenance medium. Cells were incubated at 37 °C for 15 h prior to being fixed with 4 % formaldehyde in PBS. They were subjected to IFA as described below.

### Immunofluorescence assay.

IFA was performed as previously reported (Kim *et al.*, 2016). Briefly, fixed cells on a confocal dish were permeabilized by 0.2 % Triton X-100, incubated at room temperature for 10 min and washed with PBS containing 0.1 % newborn calf serum (PBS-NCS). The mAb against SV-A capsid protein (1 : 40 dilution) was added and incubated at 4 °C overnight. Cells were then washed three times with PBS-NCS. FITC-conjugated secondary antibody (diluted to 1 : 100) was then added. Nuclei were stained with propidium iodide. Cells were then examined by confocal microscopy.

### SV-A qRT-PCR.

SV-A genome RNA levels in the faeces, sera and each organ or tissue were quantified by qRT-PCR as described previously with slight modifications (Chen *et al.*, 2014; Park *et al.*, 2013). Briefly, all tissues and fluid samples collected from experimental animals were individually weighed, homogenized or vortexed at a 1 : 10 dilution in 0.01 M PBS and centrifuged (tissues 13 000 ***g*** for 3 min; faecal samples 5000 ***g*** for 10 min). The supernatants, along with the remaining bulk samples, were collected and stored at −80 °C for analysis. To quantitate SV-A genome copy numbers, cells were infected without or with SV-A strain KS04105 at an m.o.i. of 1 p.f.u. cell^−1^ and incubated at 37 °C for 4, 8, 15 and 72 h post infection as described above. Each infected cell culture was freeze-thawed three times, and cell debris was spun down at 2469 ***g*** for 10 min at 4 °C. After extracting total RNA from supernatants, each real-time RT-PCR was performed using a Rotor-Gene real-time amplification system (Corbett Research) and SensiFAST SYBR Lo-ROX One-Step mix (Enzynomics) in a final volume of 20 µl containing 10 µl of SensiFAST SYBR Lo-ROX One-Step mix (Enzynomics), 0.2 µl of reverse transcriptase, 0.4 µl of RiboSafe RNase Inhibitor, 0.8 µl of PSV1 primer (GGCAGTAGCGTGGCGAGC at positions between 153 and 170 of the 5′-UTR), 0.8 µl of PSV2 primer (CTACTCTCCTGTAACCAGT at positions between 242 and 260 of the 5′-UTR), 4 µl of template and 3.8 µl RNase-free dH_2_O. Reverse transcription was carried out at 42 °C for 15 min followed by the activation of Hot Start DNA polymerase at 95 °C for 2 min and 45 cycles of 95 °C for 10 s, 60 °C for 14 s and 72 °C for 10 s. Quantitation of virus RNA copies was carried out using a standard curve derived from 10-fold serial dilutions of *in vitro* transcripted cRNA amplified in separate PCR tubes. Rotorgene 6000^®^ (Corbett Research) software was used to calculate the amount of SV-A-specific RNA in the samples. The threshold was defined automatically in the initial exponential phase, reflecting the highest amplification rate. With regard to the crossing points resulting from the amplification curves and threshold, a direct relation between cycle number and log concentration of RNA molecules initially present in the RT-PCR was evident. By linear regression analysis of these data, Rotorgene 6000^®^ software was used to set up a standard curve to determine the concentration of RNA present in the samples.

### Ethics statement.

All animals were handled in strict accordance with good animal practices as described in the NIH Guide for the Care and Use of Laboratory Animals (NIH Publication No. 85-23, 1985, revised 1996). Our experiment protocol was approved by the Committee on Ethics of Animal Experiments, CNU with permit number of CNU No. 2012-87.

### Statistical analysis.

All statistical analyses were performed using SPSS version 11.5.1 for Windows (SPSS). One-way ANOVA was used to determine the statistical significance (*P*<0.05).
